# Multidisciplinary biopsychosocial rehabilitation for chronic low back pain: the need to present minimal important differences units in meta-analyses

**DOI:** 10.1186/s12955-018-0924-9

**Published:** 2018-05-15

**Authors:** Silvia Gianola, Anita Andreano, Greta Castellini, Lorenzo Moja, Maria Grazia Valsecchi

**Affiliations:** 1 Clinical Epidemiology Unit, I.R.C.C.S. Orthopedic Institute Galeazzi, via R Galeazzi 4, Milan, Italy; 2 Center of Biostatistics for Clinical Epidemiology, School of Medicine and Surgery, University of Milano-Bicocca, Monza, Italy; 3 Department of Biomedical Sciences for Health, University of Milan, Milan, Italy

**Keywords:** Rehabilitation, Meta-analysis, Responsiveness, Low back pain, Patient outcome assessment

## Abstract

**Background:**

The results of meta-analyses are all too often elusive, making it difficult to interpret their relevance for clinical practice. Reporting them in minimal important difference (MID) units could improve the interpretation of evidence in meta-analyses. The aim of this study was to compare, via calculation of MID units, outcomes after multidisciplinary biopsychosocial rehabilitation (MBR) versus usual care for pain relief in chronic low back pain (LBP).

**Methods:**

We re-analyzed the data of a published Cochrane review on MBR. To attribute a MID to each pain instrument, we first searched the literature for MIDs. The MID was imputed for instruments without an established MID. We compared outcomes after MBR versus usual care for chronic LBP in the short (< 3 months), mid (> 3 and < 12 months), and long (≥12 months) term. The results of the meta-analyses are reported in MID units and interpreted as follows: if the overall effect size was greater than 1, many patients gained clinically important benefits, if it lay between 0.5 and 1.0, an appreciable number benefited, and if it fell below 0.5 few did.

**Results:**

Improvement in back pain was observed in an appreciable number of patients in the short- and medium-term after MBR: the MID was lower but still close to 1 (0.75 and 0.86 MID units, respectively). MBR probably had little or no benefit for the majority of patients in the long-term, where the MID approached 0 (0.27 MID units, confidence interval 0.07–0.48).

**Conclusions:**

Meta-analyses expressed in MID units may offer better insight into the clinical relevance of MBR: the intervention is highly recommended for reducing pain in the short- and medium-term but cannot be recommended for long-term pain reduction since the benefit decays rapidly.

**Electronic supplementary material:**

The online version of this article (10.1186/s12955-018-0924-9) contains supplementary material, which is available to authorized users.

## Background

Mechanical low back pain (LBP) is the musculoskeletal disorder with the highest prevalence in the adult population [1], and it carries considerable disability and costs for society [2]. The chronic progression of LBP is often considered a biopsychosocial problem characterised by a combination of physical, psychological and social dysfunctions [3] that are typically patient-reported and have a subjective nature. There are many different therapeutic interventions for chronic LBP, but none of them is universally accepted as the magic bullet [4]. A recent review [5] reported that non-pharmacological therapies are more common for the treatment of chronic LBP than for acute LBP [6]. One of the most frequently proposed treatments is multidisciplinary biopsychosocial rehabilitation (MBR). MBR is based on the biopsychosocial model, where health and illness are determined by a dynamic interaction between biological (genetic and biochemical), psychological (mood, personality, and behavior), and social factors (cultural, familial, socioeconomic, and medical assistance) [7].

Neuroimaging studies have shown that brain regions activated by nociceptive stimuli can also be affected by emotional and behavioral states [8]. Chronic LBP involves central sensitization, a neuropathic pain component, and may induce maladaptive coping strategies and depression [9] in which the effect of the pain becomes more complex, being both a health and a social problem that requires comprehensive care through a multidisciplinary health care team [5]. In this context, the objective of MBR is to improve physical function and modify beliefs and attitudes by addressing psychological issues or targeting social and work-related behaviour.

Meta-analysis (MA) of randomized controlled trials (RCTs) is considered the best approach to identify the actual benefit of a health intervention. A plethora of different instruments are employed to measure rehabilitation outcomes, often using continuous scales, according to the preference of the researchers who designed the study protocol [10–12]. When studies assess the same outcome but measure it differently, the summary statistic usually adopted for meta-analyses is the standardized mean difference (SMD). This metric is obtained for each study by dividing the mean differences between the intervention and the control group by the pooled standard deviation of the outcome [13]. This approach has two drawbacks: first, the effect of the same magnitude will appear different if the study populations are heterogeneous [13]; second, the effect size expressed in standard deviation units is difficult for most health professionals to interpret [14]. In addition, due to the subjective nature of the outcome variables, the cumulative estimate of the treatment effect needs to be presented as a clinically relevant measure in order to illustrate the benefit of the intervention to patients.

To overcome these limitations, the minimal important difference (MID) can be adopted as the summary statistic. The MID is defined as “the smallest difference in score in the outcome of interest that informed patients or informed proxies perceive as important, either beneficial or harmful, and which would lead the patient or clinician to consider a change in the management.” [15] Reporting study results in MID units instead of standard deviation (SD) units for individual studies, and consequently for the pooled effect, can provide a uniform metric, bypassing the issues related to the use of SMD and facilitate the interpretation of results [16].

## Aim

Our aim was to compare via meta-analyses in MID units the effects of MBR versus usual care. To do this, we re-analysed the data from a Cochrane review on “Multidisciplinary biopsychosocial rehabilitation for chronic LBP” [17]; the results are reported in MID units and the implications of this approach versus the traditional one are discussed. We selected this review as a case study because it addresses a relevant health problem in rehabilitation and because Cochrane reviews use a standardised methodology, making them an optimal source for informed choice in health care [18].

## Methods

### Case study meta-analysis

Using the data from the Cochrane review by Kamper and colleagues [17], we analyzed the summary effects for the following comparison: “MBR versus usual care in chronic LBP populations” in the short (< 3 months), medium (> 3 months and < 12 months), and long (≥12 months) term. MBR was defined as an intervention involving a physical component (e.g., an exercise program) and at least one other element from the biopsychosocial model that is psychological, social or occupational (e.g., educational psychological counselling) [17]. Usual care consisted of routine treatments: medication prescription (e.g., analgesics) [19–25]; any type of treatment, including routine physiotherapy and/or alternative medicine and/or pain medications [26–30]; medical care as directed by a medical specialist [31–33].

We focused on perceived pain, the most common patient-reported outcome in low back pain rehabilitation [10], which is variously measured across studies, with some using the visual analogue scale (VAS), the numerical rating scale (NRS), or the Short Form 36 (SF-36) Body Pain Index, and others not reporting the instrument.

We updated the case study meta-analysis and ran the original search strategy [34] in the MEDLINE database from February 2014 until December 2017.

### Literature search for anchor-based MID values and imputation of missing MID

Anchor-based and distribution based methods can be employed to establish a MID for an outcome measure in a defined population, however, there is no consensus on which technique is most appropriate [35, 36]. We chose anchor-based methods because they rely on an external indicator, i.e., a clinical variable different from the outcome for which a recognized clinical difference already exists. This approach provides a stable MID that can be used with confidence across different studies, whereas distribution-based methods, since they use statistical parameters associated with an instrument in a particular population, may not be valid in a different population [37–39].

We performed an extensive literature search to detect the smallest worthwhile effect and find an established MID for all available pain instruments related to perceived back pain in studies on nonspecific LBP (i.e., excluding specific causes such as cauda equina syndrome). We updated the search strategy adopted by Ferreira et al. in 2012 [40] who conducted a broad review of methods for determining the MID of interventions for LBP, which was updated up to May 2011 (Appendix 1). Using the same terms, we searched three electronic databases MEDLINE, Cumulative Index to Nursing and Allied Health Literature (CINAHL), and Embase from May 2011 to December 2015.

However, since clinical relevance depends on the instrument used and the trials used several different instruments, we had to deal with multiple pain instruments and their MIDs. Also, we found that not all the instruments have an established anchor-based MID. One option to address this problem in our case study meta-analysis [17] could have been to exclude them from the MA and pool only the RCTs that reported an instrument with an established anchor-based MID. However, because this would have limited the power of the MA and potentially introduced a selection bias, we adopted the distribution-based method proposed by Johnston [41] to obtain a sensible MID for the instruments for which an anchor-based MID was unavailable. The same approach was used for studies not reporting the pain measurement tool.

### Data extraction and statistical analysis

Two authors, both experienced in systematic review methodology, independently re-extracted the data (i.e., mean difference and standard deviation) from the primary studies for each comparison and abstracted the pain measurement instrument (e.g., VAS) from the studies included in the previous Cochrane review [17]. We performed the meta-analyses in MID units and also in SMD for comparison. All effect sizes are reported with their corresponding 95% confidence interval (CI). The mean difference was calculated as control minus treatment in all meta-analyses, i.e., a positive MID or SMD favors MBR over usual care.

To obtain a valid MID for the instruments lacking an anchor-based one, we first calculated the standard deviation ratio (SDR) of the studies with an anchor-based MID, i.e., the anchor-based MID of the instrument divided by the baseline standard deviation for the control group or, if not reported, the standard deviation at the end-of-treatment for the same group [16]. For each study that used an instrument without an anchor-based MID, we multiplied the SD (baseline or the end-of-treatment) by the median SDR of the studies with an anchor-based MID [16].

Next, we calculated MID units as the mean difference of individual studies divided by the MID established for the *j* instrument used in the trial and then pooled them. According to Johnstone, results expressed in MID units can be interpreted as follows: if the overall effect size is greater than 1, many patients are likely to gain clinically important benefits from the treatment; if it lies between 0.5 and 1.0, an appreciable number will benefit; and if it falls below 0.5 MID units, few may achieve important benefits [16].

The use of MID units makes it easier for clinicians to understand the effects of an intervention since they are more confident with clinical scales, such as the NRS, than with non-clinical measures such as SMD. In fact, a pooled estimate expressed in MID units can be easily converted between scales by multiplying it by the established MID of a given instrument. For example, the established MID for NRS is 2 points. By multiplying the pooled estimate in MID units by 2 points, we obtain the effect size in NRS units. All analyses were performed using R software [42].

## Results

### Selection of the MID for each instrument

We found 22 studies reporting an anchor-based MID for pain measurement instruments in musculoskeletal back pain (Additional file 1: Table S1): 14 used a NRS, 5 used a VAS, and one study each used the Pain Self-Efficacy Questionnaire (PSEQ), the Patient-Specific Functional Scale (PSFS), and the 11-Face Faces Pain Scale [17].

For the studies that reported an anchor-based MID for VAS and NRS, we used the minimal important change values proposed by Ostelo et al. [43] because they were expressed by a consensus expert panel and because they were consistent with the MID values retrieved in all the other studies found. Accordingly, we assumed an anchor-based MID of 15 for VAS and of 2 for NRS. The MIDs for the other instruments were not employed, since they were not used in the trials in our case study meta-analysis. We found no study that reported an anchor-based MID for the remaining instruments (i.e., SF-36 Body Pain Index) in low back pain.

### Descriptive characteristics

The mean change in pain from baseline in the MBR and the usual care groups at short-, medium-, and long-term follow-up are reported in Additional file 2: Table S2. Nine studies were reported on short-term follow-up after MBR versus usual care for reduction of back pain, 6 on medium-term, and 7 on long-term (Table 1).

**Table 1:** Pain measurement instruments and follow-up period

Tool	Short term (*n* = 9)	Medium term (*n* = 6)	Long term (*n* = 7)
NRS	2	2	3
VAS	4	3	4
SF-36 Body Pain Index	2	1	0
Not reported	1	0	0

Overall, the three case study meta-analyses included 13 trials. Ten RCTs employed two widely used disease-specific pain instruments: the NRS and VAS. Both instruments have demonstrated their validity and responsiveness in various settings [44]. Another valid pain instrument [45] reported in two trials [20, 21] was the SF-36 Body Pain Index. One trial did not report the instrument employed [19]. We refer the reader to the published Cochrane review for details on the descriptive characteristics of the trials [17].

### Imputation of the MID for instruments without an anchor-based MID

The distribution of the SDR for each instrument with an anchor-based MID is presented in Table 2. In the meta-analysis of the short-term period, the overall median SDR was 0.83, which we used to calculate the distribution-based MIDs for the three studies that used an instrument without an established MID. The imputed MID was: 24.8 for Tavafian 2008 [21] (using SF-36 Body Pain Index, range 0–100); 19.5 for Tavafian 2011 [20] (SF-36 Body Pain Index); 2.6 for Moix 2003 [19] (no scale reported). In the medium-term comparison, the overall median SDR was 0.79. The imputed MID was 20.4 for Tavafian 2011 [20] (SF-36 Body Pain Index). All studies had an established MID in the long-term comparison.

**Table 2:** Distribution of SDR for each instrument with an anchor-based MID

MBT vs Usual Care	Median SDR	Minimum SDR	Maximum SDR
Short-term (studies with established MID, *n* = 6/9)			
NRS (*n* = 2)	1.256	1.053	1.460
VAS 0–10 cm (*n* = 4)	0.698	0.638	0.938
Overall (*n* = 6)	0.826	0.638	1.460
Medium-term (studies with established MID, *n* = 5/6)			
NRS (*n* = 2)	0.931	0.909	0.952
VAS 0–10 cm (*n* = 3)	0.682	0.622	0.789
Overall (*n* = 5)	0.789	0.622	0.952
Long-term (studies with established MID, *n* = 7/7)			
NRS (*n* = 3)	0.909	0.769	0.952
VAS 0–10 cm (*n* = 4)	0.732	0.560	1.071
Overall (*n* = 7)	0.769	0.560	1.071

### Results of meta-analyses in MID units and comparison with results from pooling SMD

**Fig. 1:**
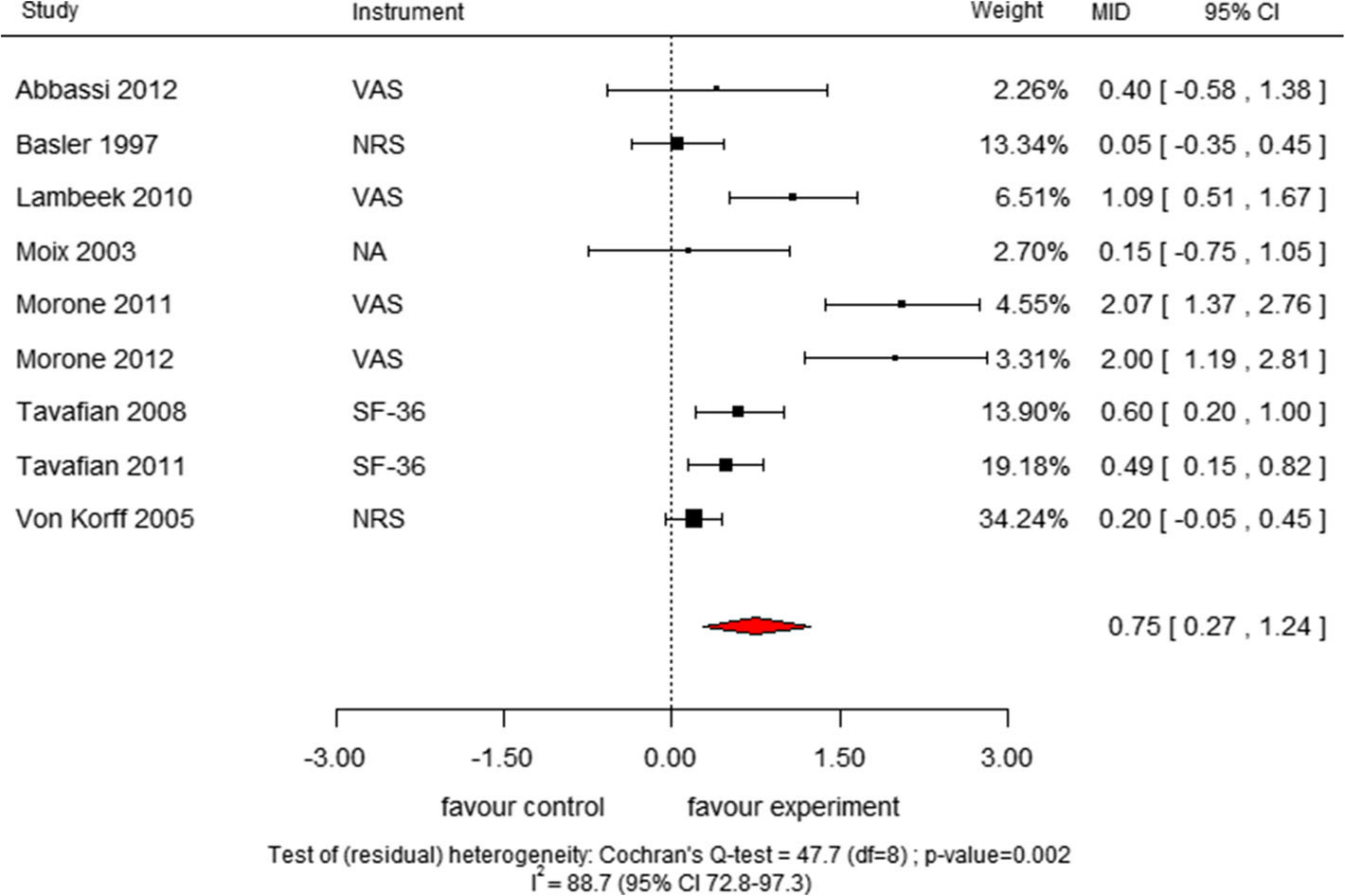
Meta-analysis of MID units for “Multidisciplinary biopsychosocial rehabilitation versus usual care for back pain in the short term”

**Fig. 2:**
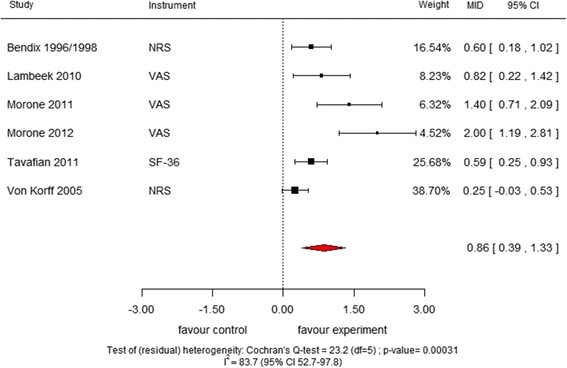
Meta-analysis of MID units for “Multidisciplinary biopsychosocial rehabilitation versus usual care for back pain in the medium term”

**Fig. 3:**
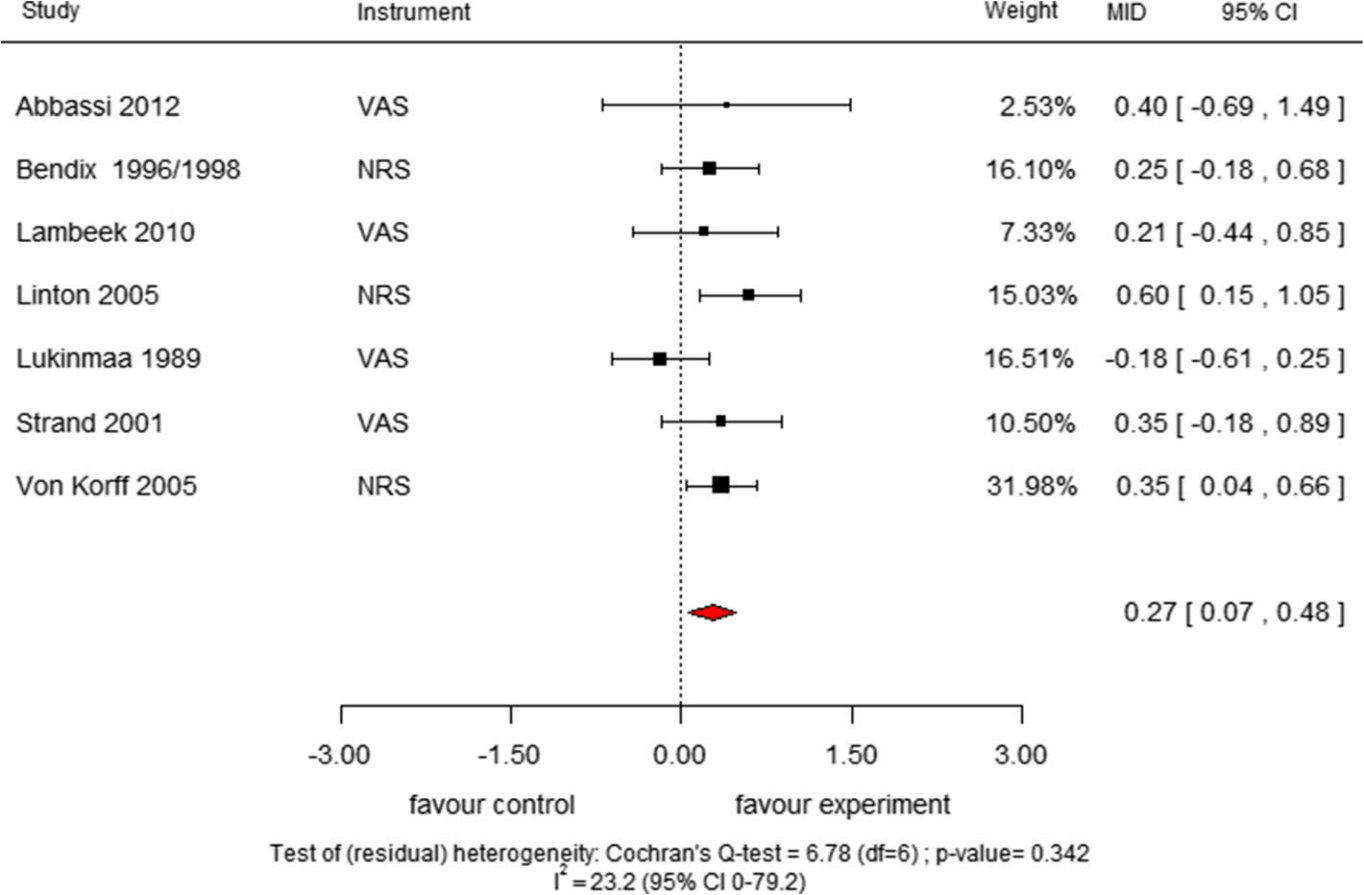
Meta-analysis of MID units for “Multidisciplinary biopsychosocial rehabilitation versus usual care for back pain in the long term”

Consistent with the Cochrane review [17], after pooling the SMDs we found a statistically significant effect in favour of MBR over usual care for pain relief in all comparisons. For the meta-analyses in MID units**,** the pooled estimate of the effect was 0.75 MID units (95% confidence interval [CI] 0.27–1.24; I^2^ 88.7, 95% CI 72.8–97.3, Fig. 1) for the short-term comparison; 0.86 MID units (95% CI 1.33–0.39; I^2^ 83.7, 95% CI 52.7–97.8, Fig. 2) for the medium-term comparison; and 0.27 MID units (95% CI 0.07–0.48; I^2^ 23.2, 95% CI 0–79.2, Fig. 3) for the long-term comparison. Given that the anchor-based established MID for NRS is 2, this finding translates into an improvement of 1.50, 1.72, and 0.54 points, respectively, on the NRS ten points scale.

**Fig. 4:**
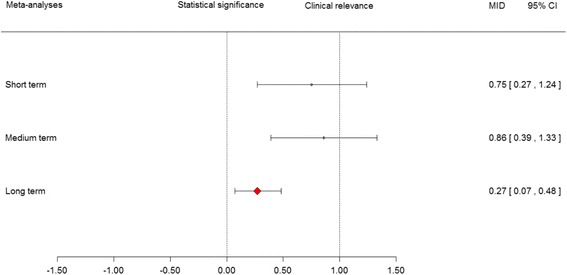
Clinical interpretation of the results of the meta-analyses in MID units

Comparison of the meta-analyses in SMD and MID units (Table 3) shows that the results are consistent across the two units of measurement: the overall estimates are statistically significant, irrespective of the approach used. From a strictly statistical point of view, we can conclude that MBR is superior to usual care at all follow-up times. All point estimates in MID units are < 1, however, i.e., the average change from baseline to the end of follow-up is smaller than the MID (Fig. 4). Also, the pooled estimates need to be carefully interpreted in light of the substantial statistical heterogeneity using the I^2^ statistics in the meta-analyses of the short- and medium-term.

**Table 3:** Meta-analyses in SMD and MID units for each follow-up period

MBR vs. Usual Care	SMD (95% CI)	MID units (95% CI)
Short-term	0.56 (0.28–0.83)	0.75 (0.27–1.24)
Medium-term	0.60 (0.34–0.87)	0.86 (0.39–1.33)
Long-term	0.21 (0.04–0.27)	0.27 (0.07–0.48)

## Discussion

We noted a limited advantage of MBR versus usual care at all follow-up times when the results of the meta-analyses were expressed in MID units. Though there was a statistically significant difference in pain relief between the two treatments, all point estimates were smaller than 1 MID, which is, by definition, the clinical sizeable benefit. Meta-analyses almost always give nominally statistically significant results at *p* < 0.05 for the difference between two treatments. This is not relevant for health care professionals, however, if the effect size is not large enough to have a practical impact on patients. Reporting MID units assumes a patient-centred perspective: treatment with a MID above 1 is expected to have important benefits for the majority of patients but for very few if below 0.5 [46]. Accordingly, comparison of MBR versus usual care for short-and medium-term relief of back pain shows that there is a real but clinically modest difference (slightly lower than 1 MID) in favour of MBR. In the long-term comparison, the benefit, albeit statistically significant, is not clinically relevant, on average, as the pooled estimated is less than one-third of 1 MID.

A systematic review published in 2007 [47] reported no effectiveness of MBR versus usual care for pain (only one of the 7 studies reported a positive effect). In contrast, the 2014 Cochrane review that we selected as case study showed that MBR, when analyzed in SMD, significantly reduced back pain in the long-term. However, the small effect and the moderate quality of evidence rated with the GRADE system led the authors to conclude that the superiority of MBR *may* be clinically relevant, a conclusion also remarked on in a recent update [34]. In our study we better quantified the clinical relevance by using the MID as a benchmark for drawing conclusions. This could be particularly relevant in the context of clinical recommendations.

According to GRADE guidelines [48], when evaluating *imprecision*, the authors of systematic reviews should consider whether the CIs of the effect size include appreciable benefit or harm [49]. Reporting meta-analyses in MID units can help readers and stakeholders judge at a glance the precision of the overall effect in terms of clinical relevance and the amount of benefit or harm against a clear anchor point. Furthermore, optimal information size can be directly related to both clinical and statistical significance. Drawing conclusions from meta-analyses based only on statistical significance may be misleading, however, especially if associated with a high prevalence of small studies and poor reporting, as is typical of the rehabilitation literature [10, 50]. There is a need to move beyond the *p*-value cliché and to focus on the magnitude of benefit since interventions of limited value sap valuable time and resources from other interventions that might have more substantial effects.

The goal of MBR is to teach individuals to cope with their pain. In doing so, the aim is to modify deeply-rooted attitudes and beliefs, as they may contribute to prolonging back pain by activating physical and emotional “triggers” [51]. It is expected that interventions will produce clinical relevant benefits in both the short- and long-term. We found no evidence of clinically relevant long-term results. One possible explication is that, after the acute phase, when the specialist sees the patient, measures baseline pain, and starts the therapy, pain may decrease over time regardless of treatment. This makes potential long-term effects smaller and more difficult to detect [52].

We showed that, in a clinically meaningful summary estimate such as the MID, the results of a meta-analysis can be interpreted differently by clinicians and patients. The main finding of our study has possible implications for recommending MBR. Multidisciplinary biopsychological rehabilitation is endorsed in the ACP/APS [53] in the National Disease Management [54] and the 2016 NICE draft guidelines [55]. Based on our results, and given the potentially high cost of MBR, these indications need to be re-considered.

The MID unit approach has some limitations in particular instances. First, the use of MID units requires that previous studies have reported an estimate of the MID (possibly an anchor-based MID) from several trials. Currently, few instruments that assess an outcome have an established MID: one study including a large cohort of trials (*n* = 185) on LBP rehabilitation found 70 different pain measurement instruments [10]. In contrast, we found only 5 instruments that had an anchor-based MID. Second, the MID is informative only about the comparison of a treatment versus a control (i.e., usual care or placebo). If we compare two different treatments, the MID value needs to be modified to account for the effect of the control treatment. For example, in the comparison of MBR versus pharmacological treatment, we should not apply the same MID that we used against usual care because the latter will already have a sizeable effect on pain relief and a smaller additional increase could be interpreted as clinically relevant. In addition, in these meta-analyses, and regardless of the unit of analysis, usual care is not the same for all studies. Also, we used a MID that does not distinguish between chronic and acute pain [43]. Finally, meta-analyses reported in MID units are vulnerable to unexperienced, oversimplified interpretation unless we keep in mind that when we define a MID, we choose a single value while, in reality, the MID is subjective, i.e., the clinical relevance of a change in outcome may be perceived differently from patient to patient.

## Conclusions

Chronic low back pain carries a poor prognosis. In such patients, a multidisciplinary rehabilitation program is believed to improve long-term pain. Performing and reporting meta-analyses in MID units proved to be useful for enhancing clinical interpretability of the results. By applying this method we were able to show a difference in the interpretation and conclusion of meta-analysis reported in SMD units: MBR has a clinically modest advantage over usual care only in the short-and medium-term follow-up. Its advantage over usual care, although statistically significant, is not clinically relevant in the long-term.

### Additional files

Additional file 1: **Table S1.** Anchor-based MID for pain measurement instruments in musculoskeletal back pain. (DOCX 49 kb)

Additional file 2: **Table S2.** Mean change in pain from baseline in the MBR and the usual care groups. (DOCX 40 kb)

## Appendix 1

MEDLINE search strategy for established MIDs.

(clinical importan* OR worthwhile change* OR important difference OR minimal clinically significant OR clinically important difference OR clinically significant difference OR clinical significance OR clinical effect* OR treatment effect* OR minimal differen* OR minim* clinically important difference OR responsive* OR “smallest detectable change” OR “minimal detectable change” OR “minimal important change” OR minim*clinical* important change OR minim* important difference OR patient expectation OR patient preference OR clinical relevance OR patient perspective OR minim* change* OR sensitivity to change OR standardized response mean) AND (“low back pain” OR “back pain” OR “spinal pain”)

## Abbreviations

CI: Confidence intervals
LBP: Low back pain
MA: Meta-analysis
MBR: Multidisciplinary biopsychosocial rehabilitation
MID: Minimal important difference
NRS: Numerical rating scale
RCT: Randomized controlled trials
SD: Standard deviation
SDR: Standard deviation ratio
SMD: Standardized mean difference
VAS: Visual analogue scale
