# Supplementary material for: Multidisciplinary biopsychosocial rehabilitation for chronic low back pain: the need to present minimal important differences units in meta-analyses
Source: Health Qual Life Outcomes. 2018 May 15;16:91. doi: 10.1186/s12955-018-0924-9 (PMC5952369; doi:10.1186/s12955-018-0924-9)
Supplement: Supplementary file 1 — Table S1. Anchor-based MID for pain measurement instruments in musculoskeletal back pain. (DOCX 49 kb) [file 12955_2018_924_MOESM1_ESM.docx]

Additional file 1: Table S1. Studies reporting an anchor based MID for pain measurement instruments in musculoskeletal back pain.

| **Outcome measure** | **Reference** | **Clinical value** |
| --- | --- | --- |
| NRS (0-10) | Childs 2005[[1](#_ENREF_1)] | 2 |
|  | Coelho 2008[[2](#_ENREF_2)] | 1,7 |
|  | Copay 2008[[3](#_ENREF_3)] | 1,2 |
|  | de Vet 2007[[4](#_ENREF_4)] | 2,5 |
|  | Farrar 2001[[5](#_ENREF_5)] | 2 |
|  | Grotle 2004[[6](#_ENREF_6)] | 1,5 |
|  | Kovacs 2007[[7](#_ENREF_7)] | 1,5 |
|  | Maughan and Lewis 2010[[8](#_ENREF_8)] | 2,4 |
|  | Ostelo 2008[[9](#_ENREF_9)] | 2 |
|  | Van der Roer 2006[[10](#_ENREF_10)] | 3.5 - 4.7 |
|  | Van der Roer 2006[[10](#_ENREF_10)] | 2.5- 4.5 |
|  | [Parker 2012](http://www.ncbi.nlm.nih.gov/pubmed/?term=Parker%20SL%5BAuthor%5D&cauthor=true&cauthor_uid=23158968)[[11](#_ENREF_11)] | 2,0-3,2 |
|  | [Carreon 2013](http://www.ncbi.nlm.nih.gov/pubmed/?term=Carreon%20LY%5BAuthor%5D&cauthor=true&cauthor_uid=23157276)[[12](#_ENREF_12)] | 1,16 |
|  | [Carreon 2013](http://www.ncbi.nlm.nih.gov/pubmed/?term=Carreon%20LY%5BAuthor%5D&cauthor=true&cauthor_uid=23157276)[[12](#_ENREF_12)] | 1,21 |
|  | [Parker 2012](http://www.ncbi.nlm.nih.gov/pubmed/?term=Parker%20SL%5BAuthor%5D&cauthor=true&cauthor_uid=22324801)[[13](#_ENREF_13)] | 2.0-6.0 |
| VAS (0-100) | Grotle 2004[[14](#_ENREF_14)] | 9,5 |
|  | Hagg 2003[[15](#_ENREF_15)] | 18,5 |
|  | Ostelo 2008[[9](#_ENREF_9)] | 15 |
|  | Sheldon 2008[[16](#_ENREF_16)] | 27,2 |
|  | [Tubach 2012](http://www.ncbi.nlm.nih.gov/pubmed/?term=Tubach%20F%5BAuthor%5D&cauthor=true&cauthor_uid=22674853)[[17](#_ENREF_17)] | 15 |
| PSEQ (pain self-efficacy questionnaire) | Maughan and Lewis 2010[[8](#_ENREF_8)] | 11 |
| PSFS (patient-specific functional scale) | Maughan and Lewis 2010[[8](#_ENREF_8)] | 1,4 |
| 11-Face Faces Pain Scale | Van Giang 2015[[18](#_ENREF_18)] | -1,7 -1,82 |

**References**

1. Childs JD, Piva SR, Fritz JM: **Responsiveness of the numeric pain rating scale in patients with low back pain.** *Spine (Phila Pa 1976)* 2005, **30:**1331-1334.

2. Coelho RA, Siqueira FB, Ferreira PH, Ferreira ML: **Responsiveness of the Brazilian-Portuguese version of the Oswestry Disability Index in subjects with low back pain.** *Eur Spine J* 2008, **17:**1101-1106.

3. Copay AG, Glassman SD, Subach BR, Berven S, Schuler TC, Carreon LY: **Minimum clinically important difference in lumbar spine surgery patients: a choice of methods using the Oswestry Disability Index, Medical Outcomes Study questionnaire Short Form 36, and pain scales.** *Spine J* 2008, **8:**968-974.

4. de Vet HC, Ostelo RW, Terwee CB, van der Roer N, Knol DL, Beckerman H, Boers M, Bouter LM: **Minimally important change determined by a visual method integrating an anchor-based and a distribution-based approach.** *Qual Life Res* 2007, **16:**131-142.

5. Farrar JT, Young JP, Jr., LaMoreaux L, Werth JL, Poole RM: **Clinical importance of changes in chronic pain intensity measured on an 11-point numerical pain rating scale.** *Pain* 2001, **94:**149-158.

6. Grotle M, Brox JI, Vollestad NK: **Concurrent comparison of responsiveness in pain and functional status measurements used for patients with low back pain.** *Spine (Phila Pa 1976)* 2004, **29:**E492-501.

7. Kovacs FM, Abraira V, Royuela A, Corcoll J, Alegre L, Cano A, Muriel A, Zamora J, del Real MT, Gestoso M, Mufraggi N: **Minimal clinically important change for pain intensity and disability in patients with nonspecific low back pain.** *Spine (Phila Pa 1976)* 2007, **32:**2915-2920.

8. Maughan EF, Lewis JS: **Outcome measures in chronic low back pain.** *Eur Spine J* 2010, **19:**1484-1494.

9. Ostelo RW, Deyo RA, Stratford P, Waddell G, Croft P, Von Korff M, Bouter LM, de Vet HC: **Interpreting change scores for pain and functional status in low back pain: towards international consensus regarding minimal important change.** *Spine (Phila Pa 1976)* 2008, **33:**90-94.

10. van der Roer N, Ostelo RW, Bekkering GE, van Tulder MW, de Vet HC: **Minimal clinically important change for pain intensity, functional status, and general health status in patients with nonspecific low back pain.** *Spine (Phila Pa 1976)* 2006, **31:**578-582.

11. Parker SL, Adogwa O, Mendenhall SK, Shau DN, Anderson WN, Cheng JS, Devin CJ, McGirt MJ: **Determination of minimum clinically important difference (MCID) in pain, disability, and quality of life after revision fusion for symptomatic pseudoarthrosis.** *Spine J* 2012, **12:**1122-1128.

12. Carreon LY, Bratcher KR, Canan CE, Burke LO, Djurasovic M, Glassman SD: **Differentiating minimum clinically important difference for primary and revision lumbar fusion surgeries.** *J Neurosurg Spine* 2013, **18:**102-106.

13. Parker SL, Mendenhall SK, Shau DN, Adogwa O, Anderson WN, Devin CJ, McGirt MJ: **Minimum clinically important difference in pain, disability, and quality of life after neural decompression and fusion for same-level recurrent lumbar stenosis: understanding clinical versus statistical significance.** *J Neurosurg Spine* 2012, **16:**471-478.

14. Grotle M, Brox J, Vøllestad N: **Concurrent comparison of responsiveness in pain and functional status measurements used for patients with low back pain.** *Spine (Phila Pa 1976)* 2004, **29:**E492-501.

15. Hagg O, Fritzell P, Nordwall A: **The clinical importance of changes in outcome scores after treatment for chronic low back pain.** *Eur Spine J* 2003, **12:**12-20.

16. Sheldon EA, Bird SR, Smugar SS, Tershakovec AM: **Correlation of measures of pain, function, and overall response: results pooled from two identical studies of etoricoxib in chronic low back pain.** *Spine (Phila Pa 1976)* 2008, **33:**533-538.

17. Tubach F, Ravaud P, Martin-Mola E, Awada H, Bellamy N, Bombardier C, Felson DT, Hajjaj-Hassouni N, Hochberg M, Logeart I, et al: **Minimum clinically important improvement and patient acceptable symptom state in pain and function in rheumatoid arthritis, ankylosing spondylitis, chronic back pain, hand osteoarthritis, and hip and knee osteoarthritis: Results from a prospective multinational study.** *Arthritis Care Res (Hoboken)* 2012, **64:**1699-1707.

18. Van Giang N, Chiu HY, Thai DH, Kuo SY, Tsai PS: **Validity, Sensitivity, and Responsiveness of the 11-Face Faces Pain Scale to Postoperative Pain in Adult Orthopedic Surgery Patients.** *Pain Manag Nurs* 2015, **16:**678-684.
