# Supplementary material for: Multidisciplinary biopsychosocial rehabilitation for chronic low back pain: the need to present minimal important differences units in meta-analyses
Source: Health Qual Life Outcomes. 2018 May 15;16:91. doi: 10.1186/s12955-018-0924-9 (PMC5952369; doi:10.1186/s12955-018-0924-9)
Supplement: Supplementary file 2 — Table S2. Mean change in pain from baseline in the MBR and the usual care groups. (DOCX 40 kb) [file 12955_2018_924_MOESM2_ESM.docx]

**Additional file 2: Table S2**. Mean change in pain from baseline in the MBR and the usual care groups.

|  | **MBR** | | | **Usual Care** | | |
| --- | --- | --- | --- | --- | --- | --- |
| **Study** | **Mean** | **SD** | **No. of patients** | **Mean** | **SD** | **No. of patients** |
| **Short term** | | | | | | |
| Abbassi 2012 | 2.6 | 2.0 | 12 | 3.2 | 1.6 | 11 |
| Basler 1997 | 4.1 | 2.1 | 36 | 4.2 | 1.4 | 40 |
| Lambeek 2010 | 3.9 | 2.5 | 60 | 5.5 | 2.3 | 62 |
| Moix 2003 | 14.5 | 3.2 | 13 | 14.9 | 3.2 | 15 |
| Morone 2011 | 4.5 | 2.3 | 41 | 7.6 | 2.1 | 29 |
| Morone 2012 | 5.0 | 2.2 | 25 | 8.0 | 2.2 | 25 |
| Tavafian 2008 | -71.5 | 16.2 | 44 | -56.6 | 30.0 | 47 |
| Tavafian 2011 | -65.8 | 22.6 | 92 | -56.3 | 23.6 | 97 |
| Von Korff 2005 | 4.9 | 2.0 | 110 | 5.3 | 1.9 | 120 |
| **Medium term** | | | | | | |
| Bendix 1996/98 | 5.7 | 2.1 | 45 | 6.9 | 2.1 | 49 |
| Lambeek 2010 | 3.6 | 2.6 | 58 | 4.8 | 2.4 | 60 |
| Morone 2011 | 4.4 | 2.5 | 41 | 6.5 | 1.9 | 29 |
| Morone 2012 | 4.0 | 2.2 | 25 | 7.0 | 2.2 | 25 |
| Tavafian 2011 | -72.3 | 22.8 | 92 | -60.3 | 25.8 | 96 |
| Von Korff 2005 | 4.2 | 2.0 | 110 | 4.7 | 2.2 | 110 |
| **Long term** | | | | | | |
| Abbassi 2012 | 3.7 | 2.5 | 12 | 4.3 | 1.4 | 11 |
| Bendix 1996/98 | 6.0 | 2.2 | 50 | 6.5 | 2.2 | 49 |
| Lambeek 2010 | 4.2 | 2.7 | 59 | 4.5 | 2.7 | 60 |
| Linton 2005 | 2.9 | 2.0 | 61 | 4.1 | 2.6 | 47 |
| Lukinmaa 1989 | 47.3 | 20.5 | 86 | 44.6 | 20.5 | 72 |
| Strand 2001 | 37.2 | 20.5 | 81 | 42.5 | 20.5 | 36 |
| Von Korff 2005 | 4 | 2.3 | 99 | 4.7 | 2.1 | 98 |
